# The role of starting knee angle in squat jump force-velocity profiles: interactions with subjects’ strength levels and imputed push-off distance

**DOI:** 10.3389/fphys.2025.1551488

**Published:** 2025-02-12

**Authors:** Xuelin Qin, Beibei Liu, Ruolin Tang, Yan Liu, Amador García-Ramos

**Affiliations:** ^1^ Department of Sport Science, Nanjing Sport Institute, Nanjing, China; ^2^ Department of Rehabilitation, Jinling Hospital, Affiliated Hospital of Medical School, Nanjing University, Nanjing, China; ^3^ Department of Physical Education and Sport, Faculty of Sport Sciences, University of Granada, Granada, Spain; ^4^ Department of Sports Sciences and Physical Conditioning, Faculty of Education, Universidad Católica de la Santísima Concepción, Concepción, Chile

**Keywords:** biomechanics, kinematics, lower-body function, testing, power

## Abstract

**Introduction:**

This study investigated whether differences in the force-velocity (F-v) profile obtained using Samozino’s method during squat jumps (SJ) performed at varying knee angles are influenced by subjects’ strength levels and the push-off distance (HpO) used in the analysis.

**Methods:**

Twenty-one resistance-trained men were classified as stronger (n = 10) or weaker (n = 11) based on the external load required to achieve a 10 cm SJ height. F-v profiles were randomly assessed over three sessions, with SJs performed at starting knee angles of 70° (SJ70), 90° (SJ90), and 110° (SJ110), using either the actual HpO specific to each condition or a standardized HpO corresponding to SJ90.

**Results:**

Significant differences between SJ types were observed for maximum force (*F*_0_) and the F-v slope (SJ110 > SJ90 > SJ70). These differences were never influenced by subjects’ strength levels. The standardized HpO produced similar outcomes for maximum power (P_max_) and maximum velocity (*v*_0_), and more consistent results for *F*_0_ and the F-v slope compared to the actual HpO.

**Discussion:**

Regardless of strength levels, standardizing HpO at 90° and allowing subjects to select a starting knee angle between 70° and 90° could simplify the evaluation process and improve the comparability of F-v profiles across subjects when using Samozino’s method.

## 1 Introduction

Vertical jumps are essential movements widely used in sports and physical performance evaluations, particularly for assessing lower-body function and muscular capabilities (McMaster et al., 2014; McMahon et al., 2019; Liu et al., 2022). Among various types of jumps, the squat jump (SJ) is extensively employed due to its simplicity, ease of standardizing the starting position, and focus on concentric muscle action (Markovic et al., 2004; Samozino et al., 2014; Fargier et al., 2016). In recent years, the assessment of the force-velocity (F-v) profile, which describes the relationship between the force applied and the velocity achieved during a movement, has gained prominence as it provides detailed insights into an athlete’s physical capacities, including maximum force (*F*_0_), velocity (*v*_0_), and power (P_max_) (Samozino et al., 2012; Cuk et al., 2014; Jaric, 2015). Such parameters are invaluable for optimizing training strategies to enhance athletic performance and may serve as critical criteria for return-to-play decisions (Morin and Samozino, 2016). For instance, Jiménez-Reyes et al. (2017) demonstrated that the F-v slope can be used to individualize training loads and exercises to maximize unloaded vertical jump performance, while Mendiguchia et al. (2016) observed that *F*_0_ is significantly altered both before and after return to sport following a hamstring injury. Consequently, analysing the impact of execution conditions—such as starting knee angles and push-off distances (HpO, the vertical displacement covered during the concentric phase of a jump)— on these mechanical outputs provides critical insights for performance evaluations and optimize training interventions.

A significant factor influencing SJ performance and the F-v profile is the knee angle at the start of the movement (La Torre et al., 2010; Argus and Chapman, 2014; Janicijevic et al., 2022; Pommerella et al., 2025). Studies have shown that varying the initial knee angle (e.g., 80°, 90°, 100°) significantly affects mechanical outputs, with larger angles (i.e., shorter HpO) typically associated with higher *F*_0_ and P_max_, while *v*_0_ remains relatively stable (Janicijevic et al., 2022; Pommerella et al., 2025). However, while previous studies have explored the influence of knee angles, the role of individual strength levels in mediating these effects has not been thoroughly investigated, representing a key research gap. Stronger individuals can generate higher force outputs, enabling them to capitalize on the extended time and displacement. In contrast, weaker individuals may struggle to leverage the extra time due to their limited force-generating capacity, potentially experiencing fatigue or inefficiency during the movement. This is supported by observations of stronger athletes achieving greater jump heights when utilizing deeper knee flexion angles during the preparatory phase of vertical jumps compared to their weaker counterparts (Ugrinowitsch et al., 2007). Investigating the relationship between starting knee angle in the SJ and its influence on the F-v profile could enable more individualized approaches to training and performance evaluation.

Within the field of biomechanics, various methods are used to evaluate F-v profiles, including force platforms, linear position transducers, and simplified approaches like Samozino’s method (García-Ramos et al., 2019). The Samozino’s method enables the calculation of force, velocity, and power outputs during vertical jumps using simple input variables such as system mass, jump height, and HpO, making it a practical and accessible tool for assessing the F-v profile (Samozino et al., 2008). Notably, HpO plays a key role in force computations; for a given system mass and jump height, smaller HpO values result in greater calculated force outputs, highlighting its critical influence on the derived mechanical parameters (Samozino et al., 2008). For instance, according to Samozino et al. (2008) equation, for a system mass of 120 kg and a jump height of 25 cm, reducing the HpO from 45 cm to 35 cm increases the calculated force from 1831 N to 2018 N, representing a 10.2% increase in force output. This observation raises an important question: could standardizing HpO—such as applying the HpO of the commonly used 90° knee angle condition uniformly across all knee angles (e.g., 70° and 110°)—yield more comparable F-v values across conditions? Standardizing HpO could simplify the evaluation process, eliminating the need for practitioners to meticulously measure HpO for each condition prior to testing. Furthermore, standardization could reduce variability and improve the comparability of results across athletes and within the same athletes across time, providing a consistent framework for interpreting performance and guiding individualized training interventions. Additionally, the 90° knee angle condition is widely recognized as both biomechanically efficient and comfortable for most athletes, making it a practical choice for standardizing assessments. In this context, athletes might be allowed to perform jumps using their self-preferred squat depth while employing a standardized HpO from a common condition over time. Such an approach is expected to ensure that athletes exhibit a single set of maximal mechanical capacities—*F*_0_, *v*_0_, and P_max_—independent of the specific conditions under which they are assessed.

Considering that the two previous studies analysing differences in F-v profiles across varying knee angles utilized the impulse-momentum relationship to compute force and velocity values (Janicijevic et al., 2022; Pommerella et al., 2025), this research aimed to determine whether similar findings could be obtained using the equations proposed by Samozino and colleagues (Samozino et al., 2008). This is important because Samozino’s method is more accessible for practitioners who may not have access to advanced equipment like force platforms. This study aimed (i) to investigate whether differences in the F-v profile obtained using Samozino’s method during SJ performed at varying knee angles (70°, 90°, and 110°) are influenced by subjects’ strength levels, and (ii) to explore whether standardizing HpO across conditions can minimize the discrepancies in F-v profiles across different starting knee angles. Our general hypothesis was that progressively decreasing the starting knee angle (i.e., shortening the HpO) would result in greater *F*_0_, higher P_max_, and steeper F-v slopes, while *v*_0_ would remain consistent across knee angle conditions (Janicijevic et al., 2022; Pommerella et al., 2025). We hypothesized that stronger individuals would show smaller differences in the F-v profile across knee angles due to their greater force-generating capacity at lower knee angles (Ugrinowitsch et al., 2007). Additionally, we expected that standardizing HpO would reduce discrepancies in F-v profiles, yielding consistent values for *F*_0_, *v*_0_, F-v slope, and P_max_, regardless of the starting knee angle, given the trivial differences in loaded SJ height across knee angles (Janicijevic et al., 2019).

## 2 Materials and methods

### 2.1 Experimental protocol

The study employed a randomized crossover design to examine how starting knee angles (70°, 90°, and 110°) influence the F-v relationship using Samozino’s method. These knee angles were chosen as they represent commonly used squat depths in both research and practical settings, covering a range of joint positions relevant for evaluating lower-body function and athletic performance. After a preliminary session, which served to familiarize participants with the SJ technique performed at different knee angles and to determine through an incremental loading test the external load associated with achieving a jump height of ≈10 cm when jumping from a 70° knee angle (i.e., the condition with the largest HpO), participants completed three testing sessions. These sessions were separated by 2–7 days to ensure adequate recovery, and participants were instructed to attend each session without fatigue. In each of the three testing sessions, only one type of SJ was evaluated (SJ70, SJ90, or SJ110), with the order of the sessions randomized to minimize potential order effects. All sessions were scheduled at the same time of day for each participant to minimize potential variations due to circadian rhythms.

### 2.2 Participants

Twenty-one resistance-trained men with prior experience in performing loaded squat jumps (SJ) participated in this study. Participants were categorized into two groups—stronger and weaker—based on their ability to perform a loaded SJ from a 70° knee angle with a minimum jump height of 10 cm under specific external loads. The stronger group (n = 10; age: 22.9 ± 2.1 years; body mass: 79.2 ± 9.6 kg; height: 178.9 ± 7.3 cm) comprised individuals who could achieve a jump height of at least 10 cm with an external load of 80 kg or more (mean load: 91.5 ± 7.8 kg; range: 80–100 kg). In contrast, the weaker group (n = 11; age: 22.9 ± 2.3 years; body mass: 73.9 ± 9.2 kg; height: 175.5 ± 5.1 cm) included participants who achieved a jump height of 10 cm with external loads ranging from 60 to 75 kg (mean load: 67.7 ± 5.6 kg; range: 60–75 kg). This classification was made arbitrarily to create two groups of similar size. A minimum load of 60 kg was established as an inclusion criterion to ensure sufficient variability and reliability in the experimental data for modelling the F-v relationship. Notably, the stability of the F-v relationship slope improves as the range between the lowest and highest experimental points increases (García-Ramos, 2023).

All participants were free from injuries or musculoskeletal conditions that could interfere with their performance during testing. They were thoroughly informed about the study’s purpose, procedures, and potential risks, and provided written informed consent prior to participation. The study protocol adhered to the ethical standards outlined in the Declaration of Helsinki and received approval from the local institutional review board.

### 2.3 Testing procedures

Each session began with a standardized warm-up, including 5 min of low-intensity cycling, self-selected joint mobility exercises, and submaximal SJ trials at progressively heavier loads using the specific knee angle assigned for that session. Participants then performed SJs under four incremental loads, which remained consistent across all conditions: (i) a light load, consisting of a 0.5 kg barbell to ensure proper loaded SJ technique, (ii) a heavy load, defined during the familiarization session as the external load that allowed a jump height of approximately 10 cm when jumping from the 70° knee angle, and (iii) two intermediate loads evenly distributed between the light and heavy loads (Janicijevic et al., 2020). For example, for a participant with a heavy load of 80 kg, the four loads were approximately 0.5 kg, 27 kg, 54 kg, and 80 kg. Rest periods of 1 min were provided between jumps with the same load and 3 min between different loads.

Each participant completed three maximal-effort trials per load, with consistent technique supervised and verified by trained researchers. They were instructed to land with feet and legs fully extended to avoid an overestimation of jump height. A chair with an adjustable bench was used to provide tactile feedback, ensuring participants reached the required knee angle for each condition. Participants did not fully sit on the chair; rather, their glutes made brief contact with the chair to confirm the correct position. After a short pause while maintaining contact, they were instructed to jump as high as possible. A tape was placed on the floor to ensure that the participants’ feet and the chair remained in the same position throughout testing. The vertical distance from the greater trochanter to the floor at the starting position was measured with a measuring tape for each SJ condition, and the HpO was calculated as the difference between the length of the fully extended lower limb and this measured distance (Samozino et al., 2014). For example, for a participant with a fully extended lower limb length of 0.97 m: in the 70° knee angle condition, if the vertical distance from the greater trochanter to the floor is 0.50 m, the HpO is 0.47 m (0.97 m–0.50 m); in the 90° knee angle condition, if the vertical distance is 0.57 m, the HpO is 0.40 m; and in the 110° knee angle condition, if the vertical distance is 0.69 m, the HpO is 0.28 m.

### 2.4 Measurement equipment and data analysis

Mean force and velocity values were calculated using Samozino’s method, which requires three input variables: system mass, jump height, and HpO (Samozino et al., 2008). Jump height was estimated from flight time using the OptoGait system (OptoGait, Microgate, Italy). The OptoGait system can underestimate jump height by approximately 1 cm (Glatthorn et al., 2011); however, this effect is expected to be consistent across the three knee angles tested. As a result, any potential inaccuracies in jump height measurements are unlikely to meaningfully affect the comparisons of F-v profiles across conditions. HpO was determined as the difference between the extended lower limb length (measured from the greater trochanter to the tip of the toes with maximal plantar flexion) and the vertical distance from the greater trochanter to the ground with knees flexed at 70° (SJ70; HpO = 47.1 ± 6.4 cm), 90° (SJ90; HpO = 39.5 ± 5.5 cm), and 110° (SJ110; HpO = 28.5 ± 4.8 cm). Note that 180° refers to full lower limb extension. The F-v relationship parameters were determined through a linear regression model, F(V) = *F*_0_ – aV, where *F*_0_ represents the force-intercept and *a* is the slope of the F-v relationship (Jaric, 2015). The velocity-intercept (*v*_0_ = *F*_0_/*a*) and maximum power (P_max_ = *F*_0_⋅*v*_0_/4) were also calculated. Mean force values were computed using either the actual HpO specific to each SJ condition or a standardized HpO corresponding to the HpO of the SJ90 condition. Consequently, five F-v relationships were modelled for each participant: (i) SJ70 using the HpO at 70°, (ii) SJ70 using the HpO at 90°, (iii) SJ90 using the HpO at 90°, (iv) SJ110 using the HpO at 110°, and (v) SJ110 using the HpO at 90°.

### 2.5 Statistical analysis

Descriptive data for the F-v relationship parameters are presented as means and standard deviations. A two-way mixed analysis of variance (ANOVA) was conducted, with SJ type (SJ70, SJ90, and SJ110) as the within-subject factor and group (stronger vs. weaker) as the between-subject factor, followed by LSD *post hoc* tests for pairwise comparisons of each F-v parameter. Only the F-v relationships using the actual HpO specific to each SJ condition were considered for this analysis. The magnitude of differences across SJ types was quantified using Hedges’ g effect size (ES), calculated as the raw mean difference divided by the pooled standard deviation of the compared conditions, with 95% confidence intervals. Effect sizes were interpreted using the following scale: trivial (<0.2), small (0.2–0.59), moderate (0.60–1.19), large (1.2–2.0), and very large (>2.0) (Hopkins et al., 2009). A two-way repeated-measures ANOVA was performed to compare the absolute differences of the F-v relationship parameters derived from the more commonly applied SJ90 with those from the other four models, considering SJ type (SJ70 vs. SJ110) and HpO type (actual vs. standardized) as within-subject factors. All statistical analyses were performed using SPSS 25.0 (IBM, Armonk, NY, United States), with significance set at *p* < 0.05.

## 3 Results

**FIGURE 1:**
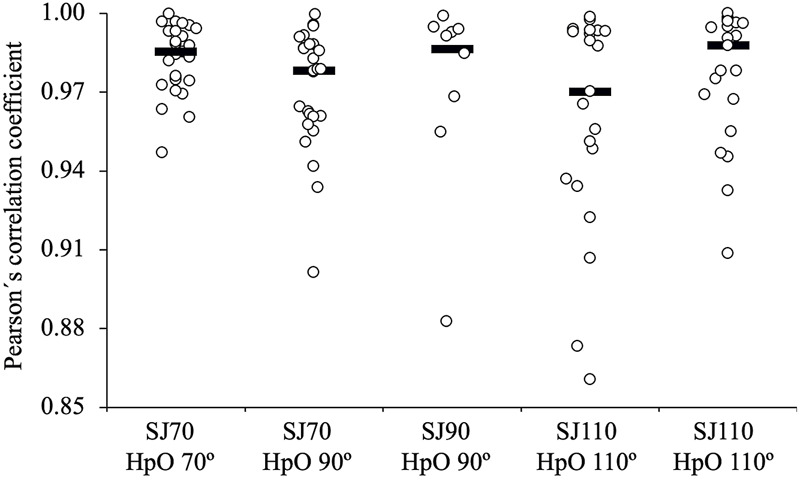
Pearson’s correlation coefficient illustrating the linearity of the individual force-velocity (F-v) relationships modelled under five different conditions: (i) SJ70 using the HpO at 70°, (ii) SJ70 using the HpO at 90°, (iii) SJ90 using the HpO at 90°, (iv) SJ110 using the HpO at 110°, and (v) SJ110 using the HpO at 90°. Each dot represents individual data points, and the bar indicates the median correlation coefficient for each condition. SJ = squat jump; HpO = push-off distance.

**FIGURE 2:**
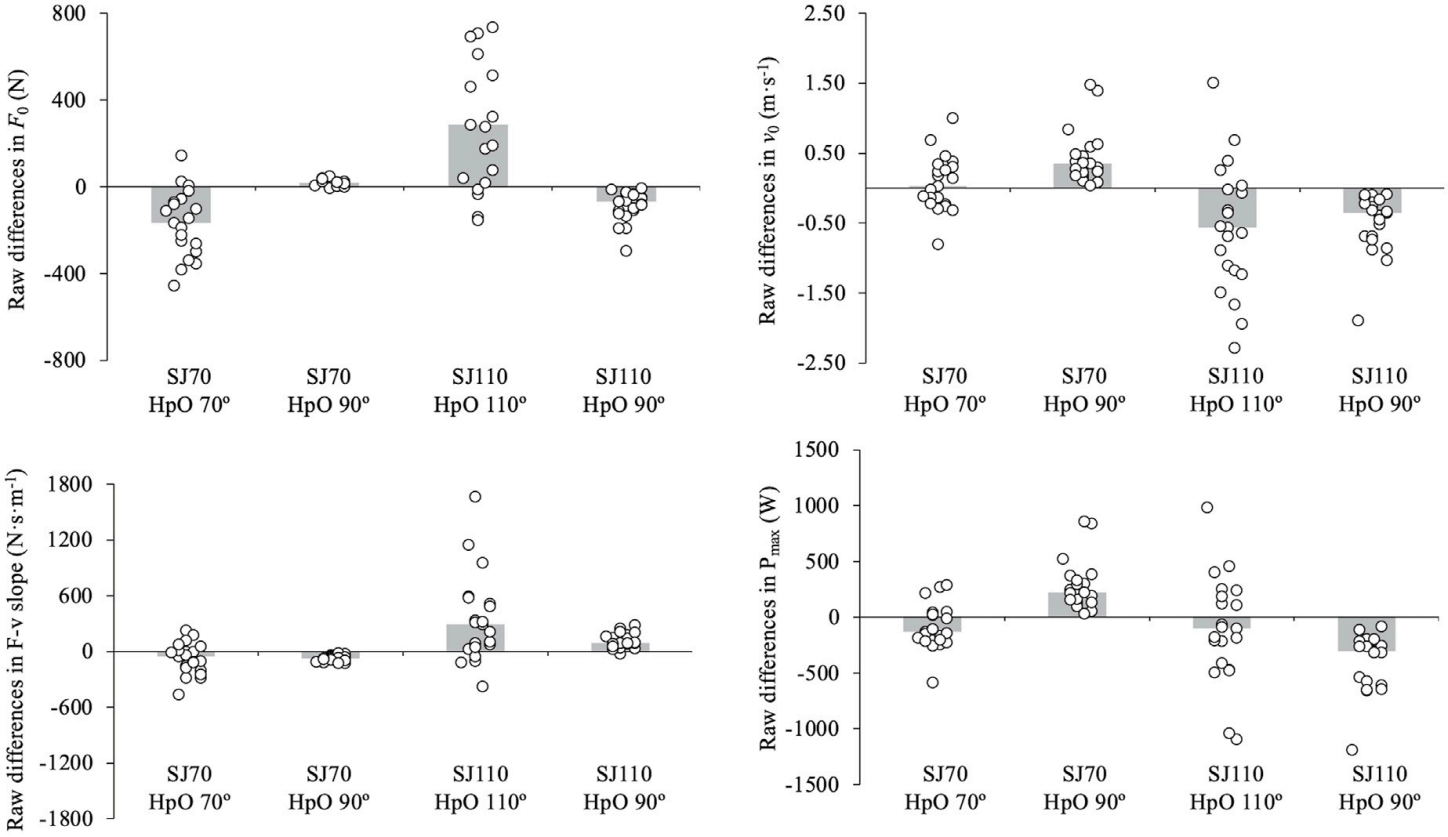
Standardized differences (Hedges’ g effect size) and 95% confidence intervals for maximum force (*F*_0_), maximum velocity (*v*_0_), force-velocity slope (F-v slope), and maximum power (P_max_) across three squat jump (SJ) condition comparisons: SJ70 vs. SJ90, SJ70 vs. SJ110, and SJ90 vs. SJ110. The mean difference is calculated as the value for the first SJ type minus the second (e.g., SJ70 - SJ90). Comparisons are presented separately for the stronger and weaker groups, with p-values and effect size (ES) reported for each parameter. Significant differences (p < 0.05) are highlighted in bold. The vertical grey band represents the range of trivial effect sizes (<0.2).

The individual F-v relationships were consistently highly linear (*r* > 0.86) (Figure 1). Significant differences between the SJ types were found only for *F*_0_ and the F-v slope, with values following the order SJ110 > SJ90 > SJ70 (Table 1). A main effect of group was observed for *v*_0_ and P_max_, as the stronger group consistently achieved greater values compared to the weaker group. However, the SJ type × group interaction never reached statistical significance. The magnitude of the differences in F-v relationship parameters across SJ types, analysed separately for the stronger and weaker groups, is illustrated in Figure 2.

**TABLE 1:** Results of the two-way mixed analysis of variance (ANOVA) comparing force-velocity (F-v) relationship parameters across squat jump types (SJ70, SJ90, and SJ110) and groups (stronger vs. weaker).

F-v parameter	SJ type	Group		ANOVA
		Stronger	Weaker	
*F*_0_ (N)	SJ70	2,627 ± 296	2,461 ± 308	**SJ: F = 12.4, *p* = 0.001** G: F = 2.9, *p* = 0.104 SJ × G: F = 0.5, *p* = 0.598
	SJ90	2,820 ± 261	2,615 ± 308	
	SJ110	3,141 ± 501	2,806 ± 557	
*v*_0_ (m·s^–1^)	SJ70	3.54 ± 0.69	2.70 ± 0.46	SJ: F = 0.5, *p* = 0.562 **G: F = 5.6, *p* = 0.029** SJ × G: F = 0.7, *p* = 0.526
	SJ90	3.33 ± 0.58	2.75 ± 0.54	
	SJ110	3.23 ± 1.31	2.71 ± 0.62	
F-v slope (N·s·m^−1^)	SJ70	782 ± 241	950 ± 268	**SJ: F = 4.6, *p* = 0.038** G: F = 0.5, *p* = 0.494 SJ × G: F = 0.6, *p* = 0.577
	SJ90	883 ± 241	1,000 ± 310	
	SJ110	1,142 ± 526	1,125 ± 545	
P_max_ (W)	SJ70	2,292 ± 318	1,648 ± 303	SJ: F = 2.5, *p* = 0.119 **G: F = 14.7, *p* = 0.001** SJ × G: F = 0.3, *p* = 0.780
	SJ90	2,325 ± 330	1782 ± 325	
	SJ110	2,421 ± 653	1865 ± 396	

Mean ± standard deviation values are reported for each condition, along with the ANOVA, results for main effects of squat jump type (SJ), group (G), and their interaction (SJ × G). *F*_0_, maximum force; *v*_0_, maximum velocity; P_max_, maximum power. Bolds values indicate significant differences (*p* < 0.05).

**FIGURE 3:**
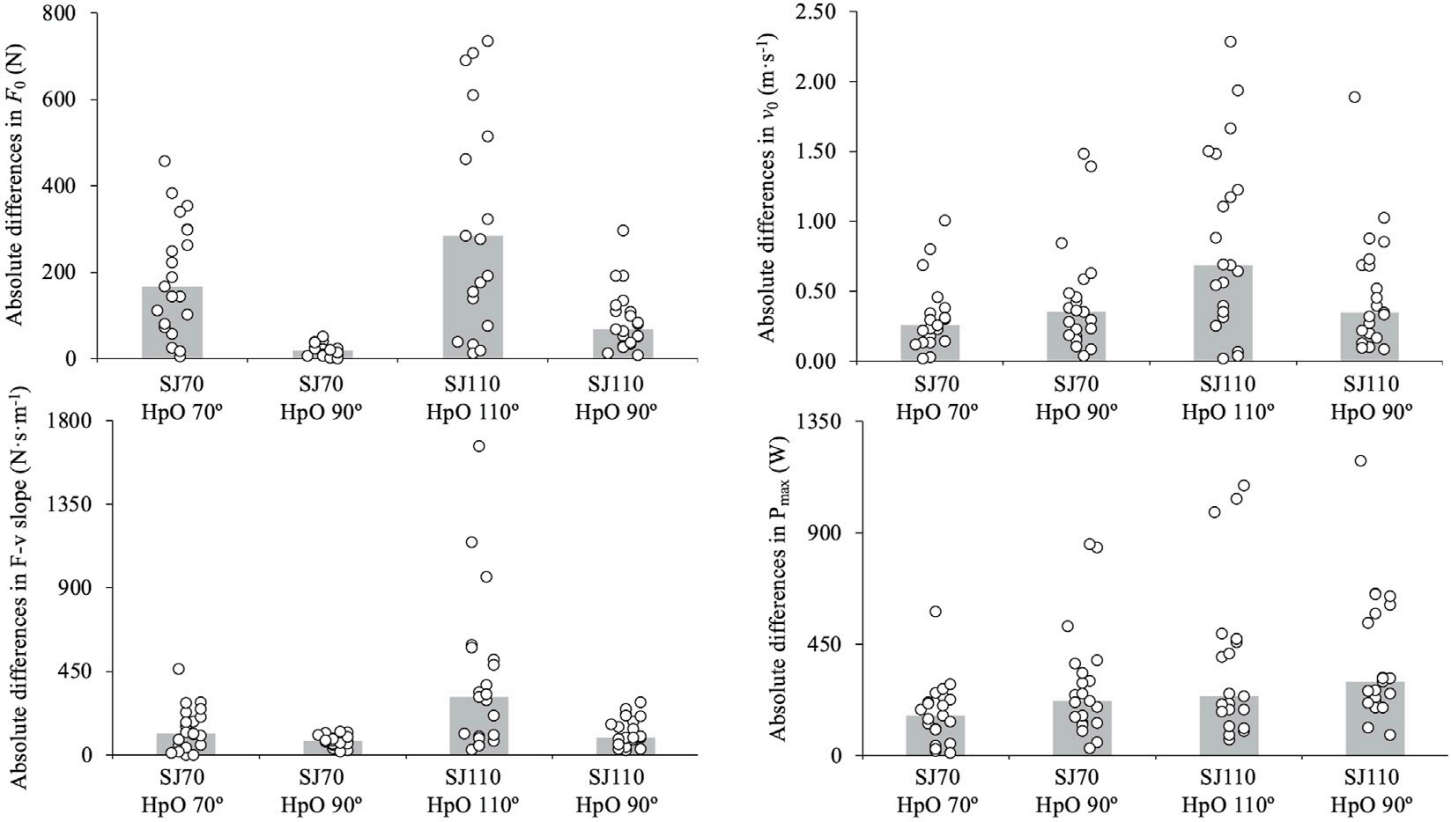
Absolute differences in force-velocity (F-v) relationship parameters—maximum force (*F*_0_; upper-left panel), maximum velocity (*v*_0_; upper-right panel), F-v slope (lower-left panel), and maximum power (P_max_; lower-right panel)—between the standard model (SJ90 with HpO at 90°) and four alternative models: (i) SJ70 with HpO at 70°, (ii) SJ70 with HpO at 90°, (iii) SJ110 with HpO at 110°, and (iv) SJ110 with HpO at 90°. Bars represent group means; individual data points are shown as open circles. SJ = squat jump; HpO = push-off distance.

**FIGURE 4:**
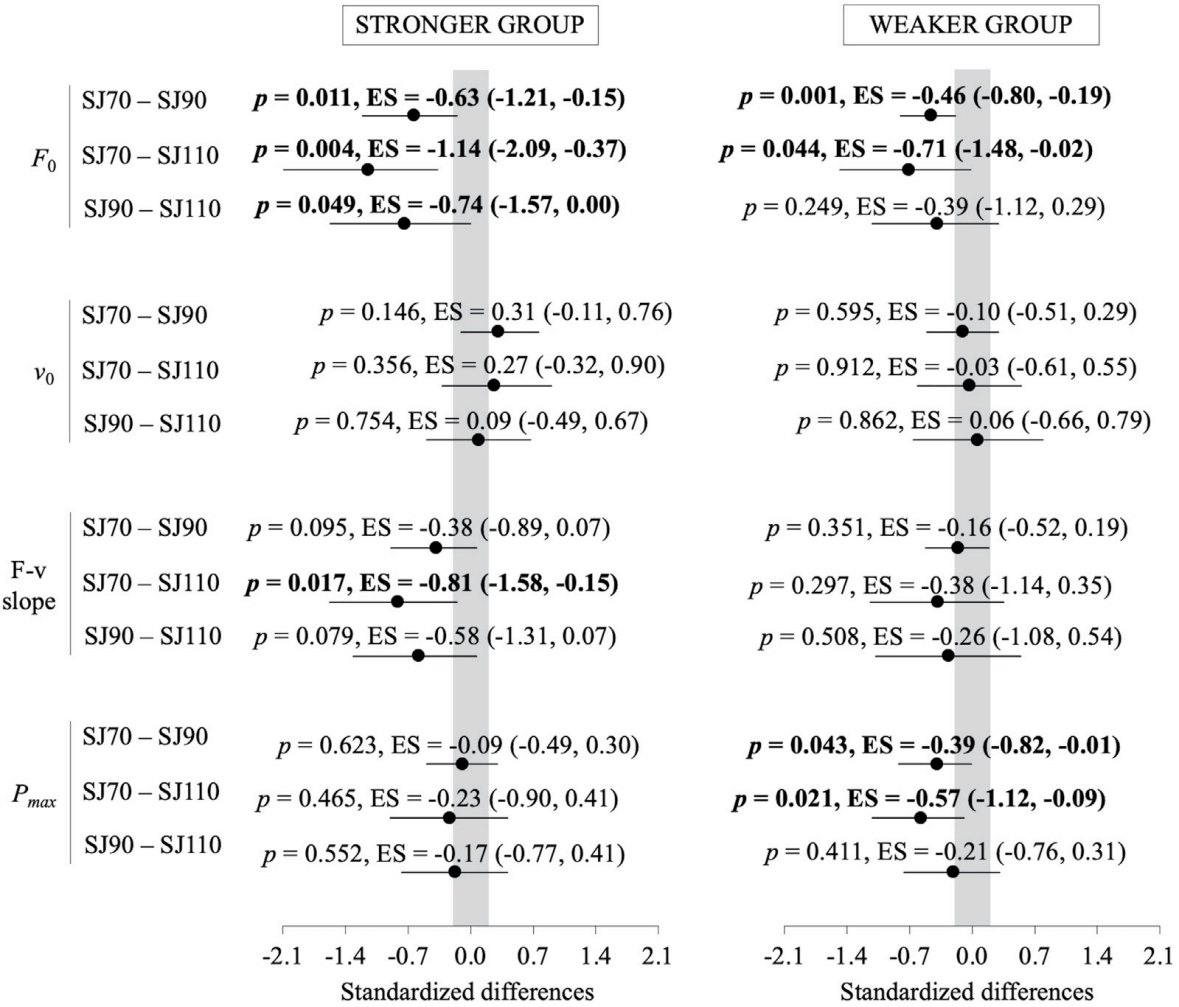
Raw differences in force-velocity (F-v) relationship parameters—maximum force (*F*_0_; upper-left panel), maximum velocity (*v*_0_; upper-right panel), F-v slope (lower-left panel), and maximum power (P_max_; lower-right panel)—between the standard model (SJ90 with HpO at 90°) and four alternative models: (i) SJ70 with HpO at 70°, (ii) SJ70 with HpO at 90°, (iii) SJ110 with HpO at 110°, and (iv) SJ110 with HpO at 90°. Bars represent group means; individual data points are shown as open circles. SJ = squat jump; HpO = push-off distance. Negative values indicate that the standard model (SJ90 with HpO at 90°) produced greater values. Bolds values indicate significant differences (*p* < 0.05).

The ANOVA applied to the absolute differences relative to SJ90 (Figure 3) revealed significant main effects for SJ type on *F*_0_ (F = 8.1, *p* = 0.010), *v*_0_ (F = 22.8, *p* < 0.001), F-v slope (F = 13.6, *p* = 0.001), and P_max_ (F = 13.5, *p* = 0.002), with greater differences observed for SJ110 compared to SJ70. Significant main effects for HpO type were found for *F*_0_ (F = 35.9, *p* < 0.001) and F-v slope (F = 9.8, *p* = 0.005), driven by larger differences when using the actual HpO versus the standardized HpO, while no significant differences were noted for *v*_0_ (F = 1.9, *p* = 0.182) or P_max_ (F = 3.6, *p* = 0.071). The SJ type × HpO type interaction was significant for *v*_0_ (F = 6.2, *p* = 0.021) and F-v slope (F = 4.5, *p* = 0.046), with greater differences for SJ110 being amplified under the actual HpO condition. The raw differences are displayed in Figures 3, 4 in the interpretation of our results in the following discussion section.

## 4 Discussion

This study aims to determine how starting knee angle and strength level influence the F-v relationship during SJ using Samozino’s method. Within this framework, we explored the influence of subjects’ strength levels (stronger vs. weaker) and the type of HpO considered (actual vs. standardized at 90°) on the resulting F-v profiles. The main findings demonstrated that while the stronger group exhibited greater *v*_0_ and P_max_ values, the primary differences between the SJ types—specifically, greater *F*_0_ and steeper F-v slope for reduced HpO—were not influenced by subjects’ strength levels. Additionally, when compared to the commonly applied SJ90 using the actual HpO, the SJ70 and the standardized HpO provided F-v relationship parameters that were more comparable, whereas the SJ110 and actual HpO introduced greater variability. These results suggest that the differences between SJ types (SJ70, SJ90, and SJ110) are independent of subjects’ strength levels. Standardizing HpO at 90° and allowing subjects to freely select a starting knee angle between 70° and 90° could be a practical approach to simplify the evaluation process and improve the comparability of the F-v relationship across subjects when using Samozino’s method.

The differences in the F-v profiles observed when performing SJs from varying knee angles align with previous studies, which found that reduced HpO leads to greater *F*_0_, higher P_max_, and steeper F-v slopes, while *v*_0_ remains relatively stable (Janicijevic et al., 2022; Pommerella et al., 2025). Our findings reaffirm the mechanical advantage of shorter HpO, as it enables greater force production by minimizing the range of motion (Kirby et al., 2011; Gheller et al., 2015; Petronijevic et al., 2018; Sanchez-Sixto et al., 2018). Notably, we observed consistent differences across SJ types using Samozino’s method, which estimates mean force and velocity values based on three input variables—system mass, jump height, and HpO. In contrast, the two previous studies investigating the effects of knee angles or push-off distances during the SJ (Janicijevic et al., 2022) and countermovement jump (CMJ) (Pommerella et al., 2025) derived mean force and velocity directly from a gold-standard force platform using the impulse-momentum relationship. Therefore, these findings demonstrate that the effects of varying push-off distances on the F-v profile are independent of the jump type (SJ or CMJ) and the analysis procedure (force platform or Samozino’s method).

This is the first study to investigate whether differences in the F-v profile of SJs performed from varying knee angles are influenced by subjects’ strength levels. Stronger individuals might benefit from larger HpO, as this provides additional time and displacement to apply force effectively during the concentric phase, while weaker individuals could experience fatigue or inefficiency under such conditions (Ugrinowitsch et al., 2007). However, our findings revealed no significant interactions between SJ type (SJ70, SJ90, SJ110) and group (stronger vs. weaker) for *F*_0_, *v*_0_, P_max_, or the F-v slope, suggesting that the differences in F-v profiles across knee angles are largely independent of strength levels. Interestingly, while the stronger group exhibited higher absolute values for *v*_0_ and P_max_, the differences in *F*_0_ were not statistically significant. Additionally, despite being classified based on the maximal external load required to achieve a 10 cm SJ height, the weaker group generally displayed a steeper F-v slope, indicating a more force-oriented profile. Although it remains unclear whether the magnitude of differences in the F-v profile across varying knee angles could be more affected by the F-v slope, the available scientific evidence indicates that the effects of varying push-off distances on the F-v profile are independent of jump type (SJ or CMJ), analysis procedure (force platform or Samozino’s method), and subjects’ strength levels.

The standardization of the push-off distance (HpO) at 90° represents a practical solution for reducing variability in the F-v relationship across different knee angles. This approach is particularly relevant because HpO is a critical factor in force computations and directly influences the mechanical outputs derived from the Samozino’s method (Samozino et al., 2008). By standardizing HpO, we observed that the F-v relationship parameters became more comparable across knee angles, especially between SJ70 and SJ90 (see Figures 3, 4). This highlights the importance of controlling for HpO to ensure consistent and reliable evaluations of an athlete’s F-v profile. Furthermore, standardizing HpO at 90° simplifies the testing process by removing the need for precise pre-measurements of HpO for each knee angle, which can be time-consuming and prone to measurement errors. This practical improvement is significant for both research and applied settings, as it enhances the feasibility and accuracy of F-v profile assessments. Notably, allowing athletes to freely select a starting knee angle within the 70° to 90° range, combined with a standardized HpO, could provide a balance between individualized performance and methodological consistency.

Despite the strengths of this study, several limitations should be acknowledged. First, the sample included only resistance-trained young men, which may limit the generalizability of the findings to other populations, such as women, untrained individuals, or older adults. However, it is worth noting that obtaining reproducible F-v profiles in individuals who cannot jump at least 10 cm with an external load of 60 kg is challenging, as increasing the distance between the lightest and heaviest experimental points is crucial for accurate modelling (García-Ramos, 2023). Therefore, the assessment of the F-v relationship during vertical jumps should be reserved for strong individuals. Second, while using a standardized HpO of 90° regardless of the starting knee angle improved the consistency of F-v profiles, this approach may result in greater discrepancies in the actual mean force values at individual loads compared to the gold-standard force platform method. Finally, the potential long-term effects of training at specific knee angles on the differences in F-v profiles across knee angles remain unexplored and warrant investigation. Future studies should aim to address these limitations by validating the standardized HpO approach in more diverse populations and settings (e.g., using the countermovement jump), as well as exploring the influence of knee angle-specific training on F-v profiles over time.

## 5 Conclusion

Decreasing the starting knee angle (i.e., shorter HpO) leads to greater *F*_0_, higher P_max_, and steeper F-v slopes, while *v*_0_ remains relatively stable. Notably, the variations in F-v profiles across knee angles were independent of subjects’ strength levels. Standardizing HpO at 90° significantly reduced variability in F-v profiles, particularly between SJ70 and SJ90. This finding highlights a practical application: by standardizing HpO at 90° and allowing subjects to select a starting knee angle within the range of 70° to 90°, practitioners can streamline the evaluation process while ensuring consistent and comparable assessments of F-v profiles across individuals using Samozino’s method. Future research should explore the long-term effects of this approach.

## Data Availability

The datasets presented in this study can be found in online repositories. The names of the repository/repositories and accession number(s) can be found below: https://osf.io/zbnds/?view_only&equals;1e7e614302d944af9360cef432607fff.
